# *γ*-Glutamyl transferase and breast cancer risk

**DOI:** 10.1038/sj.bjc.6605719

**Published:** 2010-06-01

**Authors:** I S Fentiman, D S Allen

**Affiliations:** 1 Research Oncology, Bermondsey Wing, Guy's Hospital, London, SE1 9RT, UK

**Keywords:** GGT, breast cancer, premenopausal, oxidative stress, carcinogens

## Abstract

**Background:**

It has been reported that there is an increased risk of cancer in individuals with elevated levels of serum *γ*-glutamyl transferase (GGT).

**Methods:**

In the Guernsey Breast Cancer Cohort Study, GGT was measured in sera from 1803 normal women. Among these women, 251 subsequently developed cancer, of whom 96 developed breast cancer.

**Results:**

After adjustment for age at entry, height, weight, age at menarche and first birth with nulliparity, there was a highly significant relationship between elevated GGT and breast cancer risk. In the highest quartile, the hazard ratio (HR) was 2.17 (95% confidence interval (CI): 1.19, 3.93). When subdivided by menopausal status, there was a reduced non-significant effect in postmenopausal women, whereas for premenopausal women in the highest quartile, HR was 3.81 (95% CI: 1.37, 10.59). Premenopausal women with serum GGT levels above the normal range had a significantly elevated HR of 4.90 (95% CI: 1.86, 12.94).

**Conclusions:**

These results suggest that premenopausal women with high normal (above median) serum GGT or elevated levels (⩽40 IU l^−1^) are at increased risk of breast cancer and might benefit from close surveillance, possibly with breast magnetic resonance imaging scans. Serum GGT may mark previous exposure to carcinogens and lead to the identification of DNA adducts involved in mammary carcinogenesis.

The enzyme *γ*-glutamyl transferase (GGT, EC 2.3.2.2) uniquely enables glutathione (GSH) catabolism by hydrolysing the *γ*-glutamyl bond between glutamate and cysteine. The enzyme is widely present on the external surface of most cells but has been particularly studied as a marker of hepatic dysfunction (Teschke *et al*, 1977). However, there is evidence that GGT is implicated in other major diseases, including cardiovascular disease (Ruttmann *et al*, 2005) and diabetes (Lee *et al*, 2004). In the Vorarlberg study, with 163 944 adults followed up for a median of 12 years, high GGT was significantly associated with risk of death from cardiovascular disease (Strasak *et al*, 2008). In a large Finnish cohort study of 20 158 individuals, within the normal range of GGT, there was a dose–response relationship between GGT levels and risk of type II diabetes in both men and women (Lee *et al*, 2004).

A series of 283 438 first attendants at the Vienna General Hospital gave blood for GGT analysis and were followed up for up to 13 years to determine all-cause mortality (Kazemi-Shirazi *et al*, 2007). For both males and females with elevated serum GGT, there was significantly increased mortality from all causes, cardiovascular disease, hepatobiliary disease and cancer. Mortality risk was particularly increased for those aged <30 years.

Recently, Strasak *et al* (2008) examined cancer incidence in relation to GGT levels among the 92 983 females participating in the Vorarlberg study. After a median follow-up of 13.5 years, 4884 cancer cases were diagnosed. The normal low level of GGT was taken as <17.99 U l^−1^ and, compared with this, there was a highly significant increase in hazard ratio (HR) for cancer incidence with increasing levels of GGT, so that among those with levels >72 U l^−1^, HR was 1.43 (95% confidence interval (CI): 1.28–1.61). In terms of site specificity, elevated GGT was associated with cancers of the digestive tract, respiratory tract, breast/female genital organs and haematopoetic system.

To investigate the relationship between GGT levels and risk of malignancy, particularly breast cancer, assays have been carried out on serum samples from 1888 women who participated in the Guernsey Breast Cancer Cohort Study, of whom 251 were subsequently diagnosed with cancer.

## Methods

The Guernsey Cohort Study has been underway since 1961 with the aim of identifying risk factors for breast cancer in a normal population (Wang *et al*, 2000). In the fourth phase of the study, which recruited volunteers between 1986 and 1990, 4714 women participated, all of whom were aged >32 years and were residents of Guernsey. They attended the Imperial Cancer Research Fund (now Cancer Research, UK) Laboratory in Guernsey and completed a detailed questionnaire with regard to their previous medical and reproductive history.

After the interview, blood pressure, height and weight were measured and then 60 ml of blood was taken (50 ml without anticoagulant and 10 ml with EDTA). After centrifugation, aliquots of both serum and plasma were prepared. Aliquots of 2 ml were stored at −20°C and subsequently assayed in batches. Serum samples were used in this study. Serum GGT was measured by JPS Laboratories (formerly London, UK) using a routine kinetic colorimetric method. The coefficient of variation was 6% during the assay period.

Assays were performed on 1888 samples that were randomly selected. However, 68 women had been previously diagnosed with cancer (excluding non-melanoma skin cancer), and a further three were found to have breast cancer during the study-screening process. These were excluded from the analysis. A further 14 samples were eliminated during the statistical analysis because of missing values in covariates. Thus, the final analysis was performed on 1803 samples.

Follow-up of the cohort is still active and information on cancer incidence is obtained regularly through pathology reports from the only pathology laboratory in Guernsey, as well as from death certificates and data from the South West Cancer Registry. The study was approved by all relevant ethics committees and the women provided written consent.

Cox proportional hazards regression models were fitted to estimate HRs for risk of any cancer (except non-melanoma skin cancer), risk of breast cancer and risk of any cancer excluding breast, using the Stata statistical package (Stata Corporation, 2003). For breast cancer, all values were adjusted for age at entry, height at entry, weight at entry, age at menarche and age at first birth with nulliparity. For other cancers, values were adjusted for age at diagnosis and weight.

## Results

The distribution characteristics of GGT in this population are shown in Table 1. Postmenopausal women had slightly but significantly higher values than premenopausal women. Thus, in the analyses by menopausal status, the quartiles used are appropriate for each group. GGT was also found to be positively and significantly associated with body mass index and weight in premenopausal women, and with weight in postmenopausal women. It was not found to be significantly associated with parity or age at first birth in either menopausal group.

The features of the 1803 participants, including the 96 subsequently diagnosed with breast cancer, are given in Table 2. The breast cancers were diagnosed between 21 months and 20 years after the blood sample was taken.

To determine whether GGT level was related to overall risk of cancer in this cohort, an analysis was conducted on all incident cancers. There were 251 cancers (breast 96, colorectal 21, lung 17, ovary 15, melanoma 14, endometrium 12, lymphoma 8, pancreas 8, oesophagus 7, stomach 4, bladder 4, cervix 4 and kidney 2). Results are shown in Table 3, which indicate that, overall, there was a significant increase in risk among those with GGT levels above the normal range. However, when the breast cancer cases were removed from the analysis, no significant relationship was observed between GGT and cancer risk.

As Table 4 shows, when GGT values were divided into quartiles, within the normal range, there was a highly significant increase in HR, which rose to 2.17 (95% CI: 1.19, 3.93) in the highest quartile. When analysed on the basis of GGT levels below and above the median, the latter group had an HR of 1.88 (95% CI: 1.23, 2.87).

Lim *et al* (2007) found a close association between GGT and obesity. We also found a significant association between GGT and weight, and as there is also a significant association between postmenopausal obesity and breast cancer risk (London *et al*, 1989), the results were re-analysed on the basis of menopausal status at the time of entry into the study. Premenopausal women were defined as those women who were still menstruating or women who had had a hysterectomy (± one ovary removed) and who were aged ⩽50 years. Postmenopausal women was defined as those women who were past natural menopause or have had both ovaries removed, or who have had a hysterectomy and were >50 years of age.

The results were paradoxical. As Table 5 shows, there was no relationship between GGT and breast cancer risk in those women who were postmenopausal at the time of entry. The effect was confined to those who were premenopausal and those in the highest quartile who had an HR of 3.81 (95% CI: 1.37, 10.59). The group with GGT levels above the median had an HR of 2.46 (1.22, 4.96). When those with GGT levels above the normal range (⩽40 IU l^−1^) were compared with those within the normal range, HR was 4.90 (95% CI: 1.86, 12.94) *P*=0.001. There was no relationship between disease interval and levels of GGT.

## Discussion

This study found a significant relationship between GGT and overall risk of cancer, which disappeared when breast cancer cases were removed from the analysis. After adjustment for known risk factors, including weight, there was an independent significant relationship between elevated GGT and subsequent risk of breast cancer. However, no effect was seen in postmenopausal women, whereas for premenopausal women, HR was 3.82 for the highest quartile. Those premenopausal women with serum GGT levels above the normal range had a significantly elevated HR of 4.90.

A limitation of the study is that we do not have information of alcohol intake among the participants. However, the blood samples were collected between 1986 and 1990 before there was a major increase in alcohol use in women.

Because this was a prospective study, with samples being taken before development of breast cancer, it is likely that GGT elevation was not a result of malignant transformation, but possibly had a role in this process. Although GGT has been implicated in malignancy, this is the first study to show the specific effect in terms of breast cancer risk.

*γ*-Glutamyl transferase catalyses hydrolysis and transpeptidation of extracellular GSH, thereby having a central role in maintaining glutathione homeostasis. GSH is an antioxidant, protecting cells against oxidative stress. Generation of the more reactive glycyl-cysteine reduces ferrous to ferric iron, thereby starting an iron redox cycling process (Stark *et al*, 1993). In addition, GSH functions as a detoxifier of drugs and carcinogens by means of the glutathione transferase gene superfamily (Hayes and Pulford, 1995). As GGT is the sole enzyme capable of cleaving the *γ*-glutamyl bond in GSH, areas of increased GGT may result in metal ion reduction and redox cycling. GSH/GGT-dependent pro-oxidant reaction has been found to modulate the transduction of proliferative/apoptotic signals (Accaoui *et al*, 2000). The hydrogen peroxide released by GGT produces an anti-apoptotic effect in U937 histiocytic lymphoma cells *in vitro* (Dominici *et al*, 2003). In a recent study of human GGT-transfected melanoma cells, Corti *et al* (2009) reported that increased levels of GGT led to higher rates of DNA damage and more oxidised bases.

Studies on human breast cancers have given mixed results, probably because menopausal status and age were not taken into account. Dawson *et al* (1979) reported significantly elevated levels of GGT in 10 invasive cancers compared with normal or non-involved peritumoural tissue, but found even higher levels in fibroadenomas. Levine *et al* (1983) compared 67 normal/benign and 108 cancers and found a gradation between benign, atypical and malignant lesions. Cancers that stained showed diffuse cytoplasmic staining. In a series of 79 invasive cancers, using a polyclonal antibody, Durham *et al* (1997) reported that 29% showed no immunoreactivity for GGT, whereas all benign lesions expressed GGT.

Lee and Jacobs (2006) measured persistent organic pollutant (POP) concentrations and GGT activity in sera from participants in the National Health and Nutrition Examination Survey. Of the six most frequently detected POPs, there was a positive association between POP and GGT concentrations within the normal range for five of the POPs. In the same population, there was also a graded association of blood levels of lead and cadmium with GGT concentrations (Lee *et al*, 2006). These results led Lee and Jacobs (2009) to hypothesise that serum GGT within the reference range was a marker of exposure to environmental pollutants. When creatinine-adjusted urinary cadmium levels were measured in a case–control study of 246 women with breast cancer and 254 age-matched population controls, there was a two-fold increase in risk of breast cancer in those women with cadmium levels in the highest quartile (McElroy *et al*, 2006). On this basis, GGT could reflect previous exposure to as yet unknown carcinogens initiating breast carcinogenesis.

It is of particular interest that GGT is associated with increased risk when the woman is premenopausal. In addition, GGT did not seem to be associated with an increased risk for other cancers in this cohort. This raises the possibility of identifying those women with high normal or elevated serum levels. In the absence of evidence of alcohol abuse, these could be selected for closer surveillance. The method of surveillance is still uncertain. Routine clinical examination has not been shown to reduce mortality from breast cancer, nor has mammography in younger women. A recent Consensus Conference on high-risk women, mostly because of their family history, advised that breast magnetic resonance imaging should be performed annually but not before the age of 25 years (Schwartz *et al*, 2008). If these findings can be replicated in a larger study, they may afford a new and inexpensive opportunity to delineate women who are at high risk of breast cancer with the hope of targeting surveillance, allowing an earlier diagnosis and thereby reducing the mortality accruing from this disease.

## Figures and Tables

**Table 1:** The distribution characteristics of GGT in the whole population and by menopausal status

	**All**	**Premenopausal**	**Postmenopausal**
*N*	1803	828	944
Minimum value	1	1	1
Maximum value	96	95	96
Median	11	9	13
Interquartile range	8–17	7–14	9–19

Abbreviation: GGT=*γ*-glutamyl transferase.

**Table 2:** Characteristics of individuals participating in the Guernsey Breast Cancer Cohort Study included in the GGT analyses

**Feature**	**Number (%)**
*Age (years)*	
32–40	59 (3)
41–50	812 (45)
51–60	485 (27)
61–70	288 (16)
>70	159 (9)
	
Weight (kg) mean±s.d.	65.1±10.9
Height (cm) mean±s.d.	160.8±6.2
BMI mean±s.d.	25.2±4.0
	
*Parity and age at first full-term delivery*	
Nulliparous	228 (13)
AFB ⩽20	243 (13)
AFB 21–30	1178 (65)
AFB >30	154 (9)
	
*Menopausal status*	
Premenopausal	828 (46)
Postmenopausal	944 (52)
Uncertain	31 (2)
	
*Age at menarche (years)*	
⩽13	1121 (62)
⩾14	682 (38)

Abbreviations: AFB=age at first full term delivery; BMI=body mass index; GGT=*γ*-glutamyl transferase.

**Table 3:** GGT and subsequent cancer (Cox regression adjusted by age and weight at entry)

	**All incident cancers**			**All cancers, except breast**		
	**All**	**Pre**	**Post**	**All**	**Pre**	**Post**
(*N*/cases)	(1803/251)	(828/88)	(944/160)	(1803/155)	(828/48)	(944/105)
*GGT in quartiles*	P=0.28	*P*=*0.21*	*P*=*0.59*	*P*=*0.24*	*P*=*0.82*	*P*=*0.29*
Q1 (lowest)	1.00	1.00	1.00	1.00	1.00	1.00
Q2	1.26 (0.88, 1.81)	1.59 (0.91, 2.79)	0.99 (0.62, 1.60)	1.15 (0.75, 1.78)	1.32 (0.66, 2.64)	0.96 (0.54, 1.69)
Q3	1.12 (0.78, 1.61)	1.72 (0.95, 3.12)	0.84 (0.53, 1.32)	0.68 (0.42, 1.11)	0.91 (0.38, 2.17)	0.61 (0.35, 1.09)
Q4	1.34 (0.96, 1.89)	1.67 (0.92, 3.01)	1.11 (0.73, 1.69)	1.05 (0.69, 1.60)	0.92 (0.40, 2.11)	0.99 (0.60, 1.63)
						
*GGT by median values*	*P*=*0.40*	*P*=*0.13*	*P*=*0.99*	*P*=*0.24*	*P*=*0.52*	*P*=*0.34*
Below median	1.00	1.00	1.00	1.00	1.00	1.00
Above median	1.11 (0.86, 1.44)	1.39 (0.91, 2.14)	0.98 (0.72, 1.35)	0.82 (0.59, 1.14)	0.82 (0.44, 1.51)	0.83 (0.56, 1.22)
						
*GGT by normal range*	***P*=0.053**	***P*=0.023**	*P*=*0.28*	*P*=*0.99*	*P*=*0.82*	*P*=*0.93*
In normal range	1.00	1.00	1.00	1.00	1.00	1.00
Above normal range	1.57 (0.99, 2.49)	2.63 (1.14, 6.05)	1.35 (0.78, 2.35)	1.00 (0.49, 2.05)	0.80 (0.11, 5.78)	1.04 (0.48, 2.24)

Abbreviation: GGT=*γ*-glutamyl transferase; Q1, first quartile; Q2, second quartile; Q3, third quartile; Q4, fourth quartile. Values in bold type represent significant *P*-values (*P*<0.05).

**Table 4:** Hazard ratio for breast cancer risk in relation to level of serum GGT (adjusted by age, age at first birth/nulliparity, age at menarche, height and weight)

	**All**	
	**HR (95% CI)**	**P-value**
*N* (cases)	1803 (96)	
		
*GGT in quartiles*		
Q1 (⩽8.0 IU l^−1^)	1.00	**0.011**
Q2 (8.1–11.0 IU l^−1^)	1.55 (0.81, 2.97)	
Q3 (11.1–14.0 IU l^−1^)	2.41 (1.33, 4.35)	
Q4 (>14.0 IU l^−1^)	2.17 (1.19, 3.93)	
		
*GGT by median value*		
Below median (11.0 IU l^−1^)	1.00	**0.004**
Above median	1.88 (1.23, 2.87)	
		
*GGT by normal range*		
In normal range (⩽40 IU l^−1^)	1.00	**0.003**
Above normal range	2.50 (1.36, 4.60)	

Abbreviation: CI=confidence interval; HR=hazard ratio; GGT=*γ*-glutamyl transferase; Q1, first quartile; Q2, second quartile; Q3, third quartile; Q4, fourth quartile. Values in bold type represent significant *P*-values (*P*<0.05)

**Table 5:** Breast cancer risk and GGT by menopausal status (adjusted by age, age at first birth/nulliparity, age at menarche, height and weight)

	**Premenopausal**	**Postmenopausal**		
	**HR (95% CI)**	***P*-value**	**HR (95% CI)**	***P*-value**
*N* (cases)	828 (40)		944 (55)	
*GGT in quartiles*				
Q1	1.00	**0.017**	1.00	0.56
Q2	2.19 (0.81, 5.90)		1.16 (0.48, 2.81)	
Q3	3.92 (1.52, 10.10)		1.59 (0.73, 3.43)	
Q4	3.82 (1.48, 9.89)		1.53 (0.71, 3.29)	
				
*GGT by median value*				
Below median (11.0 IU l^−1^)	1.00	**0.003**	1.00	0.19
Above median	2.68 (1.40, 5.15)		1.46 (0.83, 2.55)	
				
*GGT by normal range*				
In normal range (⩽40 IU l^−1^)	1.00	**0.001**	1.00	0.11
Above normal range	4.90 (1.86, 12.94)		1.91 (0.86, 4.25)	

Abbreviation: CI=confidence interval; HR=hazard ratio; GGT=*γ*-glutamyl transferase; Q1, first quartile; Q2, second quartile; Q3, third quartile; Q4, fourth quartile.

Quartiles for GGT by menopausal status as given below Table 2.

Median value for premenopausal=9 IU l^−1^, for postmenopausal=13 IU l^−1^. Values in bold type represent significant *P*-values (*P*<0.05)
